# The risk of antibiotics and enterocolitis for the development of inflammatory bowel disease: a Japanese administrative database analysis

**DOI:** 10.1038/s41598-022-11646-2

**Published:** 2022-05-09

**Authors:** Yosuke Shimodaira, Kenta Watanabe, Katsunori Iijima

**Affiliations:** Department of Gastroenterology and Neurology, Akita University Graduate School of Medicine, 1-1-1 Hondo, Akita City, 0108543 Japan

**Keywords:** Inflammatory bowel disease, Crohn's disease, Ulcerative colitis

## Abstract

Previous studies have shown that antibiotic use and enterocolitis increase the risk of developing inflammatory bowel disease (IBD) in western countries. However, these risk factors have not yet been identified in Asian populations. This study aimed to investigate the risk of IBD development associated with antibiotic use and enterocolitis in Japan. A Japanese health insurance claims database was used to identify patients recently diagnosed with Crohn’s disease (CD) and ulcerative colitis (UC) along with five matched participants without IBD. Episodes of antibiotic use and enterocolitis for 1 and 2 years before the date of diagnosis were analyzed using a conditional regression test. A total of 371 patients with CD and 2420 with UC were included. The adjusted odds ratio (AOR) increased in association with antibiotic use to 1.61 (95% confidence interval [CI] 1.26–2.05) and 1.20 (95% CI 1.09–1.31) and enterocolitis to 3.40 (95% CI 2.60–4.44) and 2.14 (95% CI 1.88–2.43) in 1 year in CD and UC, respectively. The risk associated with antibiotics was independent of the number or type of antibiotics, and the risk associated with enterocolitis did not differ with the pathogen that caused the disease. However, prior exposure to antibiotic use and enterocolitis was associated with an increased risk of developing IBD.

## Introduction

Inflammatory bowel disease (IBD), comprising Crohn’s disease (CD) and ulcerative colitis (UC), is characterized by chronic intestinal inflammation of unknown causes that results from an inappropriate intestinal immune response to luminal contents in genetically susceptible individuals^1^. The prevalence of IBD is higher in western countries than in other regions but is increasing globally^2^, and it has been estimated that more than 6–8 million people suffer from IBD worldwide^3^. A nationwide survey in Japan showed that approximately 70,000 and 220,000 people were estimated to be affected by CD and UC, respectively^4^. Moreover, since the incidence and prevalence of IBD are increasing^2^, understanding the etiology and prevention of IBD is crucial for mitigating its effects.

Host-microbe interactions are crucial for the development and modulation of the intestinal immune system^5,6^. Multiple studies have found that the pathogenesis of IBD requires a dysregulated intestinal immune response to microbes. The loss of intestinal microbiota diversity is evident in patients with IBD. Certain bacteria have been associated with IBD, such as *Faecalibacterium prausnitzii* of the phylum Firmicutes, which were found to be reduced in IBD^7,8^ and some Clostridia strains, which were found in decreased levels in fecal samples of patients with IBD^9^. These observations indicate the capacity of gut microbiota to affect IBD pathogenesis; however, whether the gut microbiome alteration is the cause or result of IBD remains controversial.

Antibiotic use and gastrointestinal infections can significantly affect the intestinal microbiome, and the effects of antibiotic use on gut microbiota likely persist over extended periods^10,11^. Several database-based analyses have revealed an increased risk of IBD development with antibiotic use^12–14^ as well as with those individuals who experienced infectious enterocolitis in western countries^15–17^. However, no similar data has been shown in Asian populations. Importantly, the genetic susceptibility of western and Asian populations varies due to differences in genetic polymorphisms. Therefore, investigating the effect of environmental factors on IBD development in Asian populations is worthwhile. To investigate the environmental risk factor associated with IBD onset, we analyzed the use of antibiotics and enterocolitis to the onset of IBD using a conditional regression test that uses real-world medical data and has a potential risk of confounding for analyses. In the present study, we accessed a large-scale database to investigate the individual effect of antibiotic use and enterocolitis on IBD development.

## Results

### Characteristics of subjects included for analysis

Table 1 shows the characteristics of 2791 participants included in this study with newly diagnosed IBD, 371 of whom were diagnosed with CD and 2420 with UC. Matched 1855 control participants to CD and 12,100 control participants to UC were identified. The age at diagnosis was 33.1 ± 15.5 years (mean ± standard deviation [SD]) for patients with CD and 40.3 ± 12.9 years for those with UC. The percentage of patients who had been prescribed antibiotics in a 3–12 month period before the diagnosis of IBD was 52.6% for patients with CD, 41.7% for patients with UC, and 35.0% for the non-IBD control participants. Antibiotic types with different mechanisms of action are shown in Table 1. Of all CD, UC or non-IBD controls, 24.0%, 25.0%, and 22.3%, respectively, were prescribed one type of antibiotic; 18.3%, 11.2%, and 9.0%, respectively, were prescribed two types of antibiotics; and 10.2%, 5.5%, and 3.7%, respectively, were prescribed three or more types of antibiotics. *Escherichia coli* enterocolitis (*E. coli*), *Campylobacter* enterocolitis (*Camp*), and *Clostridioides difficile* enterocolitis (*CDE*) including pseudomembranous enterocolitis, were included for bacterial pathogens specific to enterocolitis, and the frequency of *E. coli* infections in CD, UC or non-IBD was 0%, 0.08%, and 0.01%, respectively. *Camp* frequency of 0.27%, 0.25%, and 0.25%, and *CDE* frequency of 0.81%, 0.37%, and 0.02% were observed in CD, UC, and the non-IBD control populations, respectively. Bacterial pathogen nonspecific enterocolitis occurred in 32.3%, 17.1%, and 8.9% of patients with CD, UC, and a non-IBD diagnosis, respectively.

**Table 1:** Cohort demographic characteristics of newly diagnosed CD and UC, and time-, age-, and gender-matched non-IBD population included in this study.

	C D	U C	All IBD	Control^a^
Overall, number	371	2420	2791	13,955
Male gender (%)	288 (77.6)	1596 (66.0)	1884 (67.5)	9420 (67.5)
**At diagnosis**				
Age, mean ± SD (years)	33.1 ± 15.5	40.3 ± 12.9	39.4 ± 13.5	39.4 ± 13.5
Age, 1st, 2nd, 3rd quartile	20, 32, 46	32, 41, 50	30, 40, 49	30, 40, 49
Hospitalized^b^, number (%)	178 (48.0)	342 (14.1)	520 (18.6)	
**Exposure, number (%)**				
Antibiotics^c^	195 (52.6)	1010 (41.7)	1205 (43.2)	4879 (35.0)
Types				
Tetracycline	11 (3.0)	51 (2.1)	62 (2.2)	206 (1.5)
Penicillin	31 (8.4)	103 (4.3)	134 (4.8)	502 (3.6)
Cephalosporin	109 (29.4)	502 (20.7)	611 (21.9)	2444 (17.5)
Carbapenem	4 (1.1)	25 (1.0)	29 (1.0)	91 (0.7)
TMP/SMX^d^	3 (0.8)	17 (0.7)	20 (0.7)	42 (0.3)
Macrolide	88 (23.7)	372 (15.4)	460 (16.5)	1872 (13.4)
Aminoglycoside	12 (3.2)	94 (3.9)	106 (3.8)	453 (3.3)
Quinolone	75 (20.2)	356 (14.7)	431 (15.4)	1469 (10.5)
Anti-fungal agents^e^	2 (0.5)	8 (0.3)	10 (0.4)	9 (0.1)
Others^f^	20 (5.4)	65 (2.7)	85 (3.1)	213 (1.5)
Frequency^g^				
1	89 (24.0)	604 (25.0)	693 (24.8)	3111 (22.3)
2	68 (18.3)	272 (11.2)	340 (12.2)	1259 (9.0)
3 +	38 (10.2)	134 (5.5)	172 (6.2)	509 (3.7)
Enterocolitis^h^				
Bacterial pathogen nonspecific^h^	120 (32.3)	415 (17.1)	535 (19.2)	1238 (8.9)
Bacterial pathogen specific^i^	4 (1.08)	17 (0.7)	21 (0.8)	7 (0.05)
*Escherichia coli*	0 (0)	2 (0.08)	2 (0.07)	2 (0.01)
Campylobacter	1 (0.27)	6 (0.25)	7 (0.25)	2 (0.01)
Clostridioides	3 (0.81)	9 (0.37)	12 (0.43)	3 (0.02)

^a^Including time-, age-, and gender-matched non-IBD population for CD and UC.

^b^Hospitalized at the month or next month with IBD diagnosis.

^c^Prescribed betwwen 3 and 12 months before IBD diagnosis.

^d^Trimethoprim/Sulfamethoxazole.

^e^Amphotericin, Imidazole, and Triazole.

^f^Metronidazole, Glycopeptide, Polymixin, Fosfomycin, Linezolid, Fidaxomicin, Rifaximin, and Anti-mycobacterials.

^g^The number of types of antibiotics prescribed.

^h^Between 3 and 12 months before diagnosis with bacterial pathogen nonspesific enterocolitis.

^i^For 12 months before diagnosis with bacterial pathogen specific enterocolitis.

### Antibiotic use and enterocolitis were associated with the development of IBD

Conditional logistic regression analysis showed increased AOR on antibiotic use with 1.61 (95% confidence interval [CI] 1.26–2.05) and 1.20 (95% CI 1.09–1.31) in CD and UC respectively (Table 2). Furthermore, the AOR for enterocolitis was 3.40 (95% CI 2.60–4.44) and 2.14 (95% CI 1.88–2.43) for patients with CD and UC, respectively. We additionally analyzed the risk of antibiotic use and enterocolitis in 2 years before diagnosis to confirm whether the risk depends on the length of the period. AOR on antibiotic and enterocolitis was 1.24 (95% CI 0.96–1.60) and 1.22 (95% CI 1.11–1.34), and 3.46 (95% CI 2.66–4.48) and 1.78 (95% CI 1.60–1.97) in CD and UC, respectively. These results established the independent risk of antibiotic use and enterocolitis for the initiation of CD and UC.

**Table 2:** AORs and 95% CIs using conditional logistic regression analysis for CD, UC, and all IBD (CD and UC combined).

	C D		U C		All IBD	
	AOR^a^ (95% CI^b^)	*P* value	AOR (95% CI)	*P* value	AOR (95% CI)	*P* value
**1 year**						
Antibiotic use	1.61 (1.26–2.05)	1.16 × 10^–4^	1.20 (1.09–1.31)	1.80 × 10^–4^	1.24 (1.14–1.35)	1.38 × 10^–6^
Enterocolitis	3.40 (2.60–4.44)	4.30 × 10^–19^	2.14 (1.88–2.43)	6.64 × 10^–31^	2.34 (2.08–2.62)	7.52 × 10^–47^
**2 years**						
Antibiotic use	1.24 (0.96–1.60)	9.92 × 10^–2^	1.22 (1.11–1.34)	2.83 × 10^–5^	1.22 (1.12–1.34)	8.05 × 10^–6^
Enterocolitis	3.46 (2.66–4.48)	9.90 × 10^–21^	1.78 (1.60–1.97)	2.86 × 10^–27^	1.94 (1.76–2.13)	7.44 × 10^–42^

^a^Adjusted odds ratio.

^b^Confidence interval.

### The number and type of antibiotics used and the onset of IBD

We next analyzed the risk associated with being prescribed variable numbers of antibiotic types using logistic regression analysis. The odds ratios determined for one, two, and three or more antibiotic types were 1.43 (95% CI 1.08–1.88), 1.66 (95% CI 1.41–1.95), and 1.52 (95% CI 1.32–1.75), respectively in CD, and 1.26 (95% CI 1.13–1.40), 1.18 (95% CI 1.10–1.27), and 1.20 (95% CI 1.12–1.28), respectively in UC (Table 3). The odds ratios associated with the type of antibiotic prescribed are shown in Table 4. Penicillin, cephalosporin, macrolide, and quinolone were determined to increase the risk of CD development in this analysis. In addition, tetracycline, trimethoprim/sulfamethoxazoles (TMP/SMXs), aminoglycoside, and anti-fungal agents were determined to increase the risk for UC.

**Table 3:** AORs and 95% CIs using logistic regression analysis, number of antibiotic types prescribed.

Antibiotic prescriptions, number of types	C D		U C		All IBD	
	AOR (95% CI)	*P* value	AOR (95% CI)	*P* value	AOR (95% CI)	*P* value
1	1.43 (1.08–1.88)	1.29 × 10^–2^	1.26 (1.13–1.40)	1.69 × 10^–5^	1.28 (1.16–1.41)	1.04 × 10^–6^
2	1.66 (1.41–1.95)	9.80 × 10^–10^	1.18 (1.10–1.27)	7.17 × 10^–6^	1.24 (1.16–1.33)	8.37 × 10^–11^
3 + ^a^	1.52 (1.32–1.75)	6.36 × 10^–9^	1.20 (1.12–1.28)	1.37 × 10^–7^	1.25 (1.17–1.32)	8.18 × 10^–13^
All^b^	1.98 (1.58–2.49)	2.68 × 10^–9^	1.35 (1.23–1.47)	8.24 × 10^–11^	1.42 (1.30–1.54)	1.62 × 10^–16^

^a^Three or more.

^b^Incorporate 1, 2, and 3 +.

**Table 4:** AORs and 95% CIs using logistic regression analysis, antibiotic type prescribed.

Antibiotic type	C D		U C		All IBD	
	AOR (95% CI)	*P* value	AOR (95% CI)	*P* value	AOR (95% CI)	*P* value
Tetracycline	2.00 (0.98–4.05)	5.55 × 10^–2^	1.44 (1.05–1.98)	2.24 × 10^–2^	1.52 (1.14–2.02)	4.41 × 10^–3^
Penicillin	2.14 (1.38–3.30)	6.06 × 10^–4^	1.22 (0.98–1.52)	7.82 × 10^–2^	1.35 (1.11–1.64)	2.46 × 10^–3^
Cephalosporin	1.94 (1.50–2.50)	3.35 × 10^–7^	1.24 (1.11–1.38)	1.34 × 10^–4^	1.32 (1.20–1.46)	4.49 × 10^–8^
Carbapenem	2.52 (0.76–8.44)	1.33 × 10^–1^	1.51 (0.96–2.37)	7.16 × 10^–2^	1.60 (1.05–2.44)	2.83 × 10^–2^
TMP/SMX^a^	3.02 (0.72–12.7)	1.31 × 10^–1^	2.31 (1.30–4.10)	4.43 × 10^–3^	2.39 (1.40–4.08)	1.37 × 10^–3^
Macrolide	1.91 (1.46–2.52)	3.31 × 10^–6^	1.18 (1.05–1.34)	7.29 × 10^–3^	1.28 (1.14–1.43)	1.92 × 10^–5^
Aminoglycoside	0.71 (0.38–1.30)	2.65 × 10^–1^	1.29 (1.02–1.62)	3.31 × 10^–2^	1.18 (0.95–1.46)	1.38 × 10^–1^
Quinolone	2.35 (1.75–3.16)	1.66 × 10^–8^	1.45 (1.28–1.64)	9.67 × 10^–9^	1.55 (1.38–1.74)	1.10 × 10^–13^
Anti-fungal agents^b^	3.35 (0.56–20.1)	1.87 × 10^–1^	6.69 (2.32–19.3)	4.39 × 10^–4^	5.57 (2.26–13.7)	1.88 × 10^–4^
Others^c^	3.73 (2.07–6.69)	1.07 × 10^–5^	1.78 (1.34–2.37)	8.02 × 10^–5^	2.03 (1.57–2.62)	5.45 × 10^–8^

^a^Trimethoprim/Sulfamethoxazole.

^b^Amphotericin, Imidazole, and Triazole.

^c^Metronidazole, Glycopeptide, Polymixin, Fosfomycin, Linezolid, Fidaxomicin, Rifaximin, and Anti-mycobacterials.

### Bacterial pathogen nonspecific and specific enterocolitis increased the risk of IBD onset

Bacterial pathogen nonspecific enterocolitis increased the risk of CD and UC development, producing an AOR of 4.04 (95% CI 3.10–5.27) and 2.22 (95% CI 1.96–2.51), respectively, and the risk of bacterial pathogen specific enterocolitis was striking with an AOR of 20.3 (95% CI 2.26–182) and 14.3 (95% CI 5.63–36.2) in CD and UC, respectively (Table 5).

**Table 5:** AORs and 95% CIs using logistic regression analysis, enterocolitis.

Enterocolitis	C D		U C		All IBD	
	AOR (95% CI)	*P* value	AOR (95% CI)	*P* value	AOR (95% CI)	*P* value
Bacterial pathogen nonspecific	4.04 (3.10–5.27)	4.50 × 10^–25^	2.22 (1.96–2.51)	2.20 × 10^–36^	2.46 (2.20–2.75)	2.83 × 10^–56^
Bacterial pathogen specific	20.3 (2.26– 182)	7.18 × 10^–3^	14.3 (5.63–36.2)	2.19 × 10^–8^	15.1 (6.42–35.6)	4.95 × 10^–10^
Enterocolitis all	4.09 (3.14–5.33)	1.47 × 10^–25^	2.25 (1.99–2.55)	5.69 × 10^–38^	2.49 (2.23–2.79)	2.26 × 10^–58^

### The risk of antibiotics and enterocolitis for IBD development in children and adults

We analyzed the risk of CD and UC with antibiotic use and enterocolitis according to age. Enterocolitis but not antibiotic use showed an increased risk of UC in populations under 18 years of age, while both enterocolitis and antibiotic use showed an increased risk of CD in children and CD and UC in adults (Table 6).

**Table 6:** AORs and 95% CIs using conditional logistic regression analysis for CD and UC.

	C D		U C	
	AOR (95% CI)	*P* value	AOR (95% CI)	*P* value
**≤ 18 years**				
Antibiotic use	1.98 (1.2–3.26)	7.63 × 10^–3^	1.34 (0.97–1.84)	7.68 × 10^–2^
Enterocolitis	3.91 (2.29–6.67)	5.74 × 10^–7^	2.38 (1.61–3.53)	1.55 × 10^–5^
** ≥ 19 years**				
Antibiotic use	1.51 (1.15–1.96)	2.66 × 10^–3^	1.18 (1.07–1.3)	1.2 × 10^–3^
Enterocolitis	3.59 (2.60–4.97)	1.16 × 10^–14^	2.1 (1.83–2.4)	2.01 × 10^–26^

## Discussion

In the present study, we evaluated the risk of antibiotic use and enterocolitis for the development of IBD using a Japanese large-scale insurance claims database. This database was recently utilized for epidemiological, real-world prescription, and disease burden data^18–20^. Several large database-dependent studies have determined that antibiotic use and enterocolitis are known risk factors associated with the onset of IBD^12–17^. However, since enterocolitis is often treated with antibiotics, an assessment that considered the confounding factors was needed to evaluate the individual risk of enterocolitis and antibiotic use. In addition, antibiotic types prescribed and the use of multiple classes of antibiotics were assessed to investigate which factors associated with antibiotic use were most impactful.

Given that several reports largely consisted of western populations, our data, which comprised Japanese populations, are important for establishing that antibiotic use and enterocolitis affect IBD development.

Genetic susceptibility of IBD in western and Asian populations varies due to differences in genetic polymorphisms. For example, NOD2 variation affects IBD in some western countries^21,22^, and NOD2 has been closely associated with the host response to intestinal bacteria^23–25^. However, the same IBD-associated polymorphisms within NOD2 do not exist in Japanese populations with similar mutations^26,27^. It was indicated that different factors that affect intestinal microbiota could be associated with IBD presentation in different genetic backgrounds. Thus, investigating the effect of antibiotics and enterocolitis on the development of IBD in Asian populations is worthwhile.

We assessed antibiotic use and enterocolitis using a conditional logistic regression model, and each factor was individually marked as a risk factor in developing IBD. The present study indicates that an excess risk of IBD development occurs due to exposure to most types of antibiotics. However, some types of antibiotics were not established as risk factors for IBD development, probably because the number of individuals included in the analysis was insufficient. A previous report that involved a Canadian cohort demonstrated that most types of antibiotics were associated with an increased IBD risk^13^. However, the risk of IBD development did not increase according to the number of unique antibiotic types prescribed. Three or more courses of antibiotics increased the risk of IBD development in children^14^ but this has not been observed in another study^13^. Collectively, data reported here and elsewhere indicate that a first-time antibiotic prescription contributes most significantly to an increased IBD risk.

Enterocolitis strongly affected IBD development, and the odds ratio showed that the risk was higher in CD than in UC. Exclusion of nonspecific enterocolitis for 3 months before an IBD diagnosis diminished the possibility that latent undiagnosed IBD was misdiagnosed as enterocolitis. Bacterial pathogen specific enterocolitis also increased the risk of IBD, despite the small number of individuals involved in the analysis. Previous reports^15,16^ also showed an elevated odds ratio in patients with IBD with a prior enterocolitis episode, and the risk was higher in CD than in UC. Consistent results of an increased risk of IBD indicate a disturbance in luminal microorganisms, which likely occurs due to the effects of infectious enterocolitis on the intestinal immune response in susceptible individuals that promote IBD, and especially CD.

A strength of this study was the incorporation of a large-scale dataset using a database in which the medical treatment of each individual included prescriptions from both outpatient services and hospitalizations. Furthermore, it was possible to pick matched non-IBD individuals included in the database who were part of the insurance association during the same calendar period and were age-, and gender-matched randomly to individuals with CD or UC. Exposure to antibiotics and enterocolitis were assessed as confounding factors. The effects of latent periods of IBD with the potential to produce reverse causation were addressed by excluding individuals who were prescribed antibiotics and diagnosed with nonspecific enterocolitis 3 months before an IBD diagnosis.

The present study design also had some limitations. One is associated with the exclusion of the prescriptions in the three-month period before IBD diagnosis. The risk of antibiotic use for CD was confirmed in one year but not significant in two years in the present data. Given that a microbial disturbance by antibiotics directly affects the host intestinal immune system, an assessment of the short-term effects of antibiotic use is necessary. However, it is difficult to clarify this important timepoint unless a diagnosis at the onset of IBD is distinguished from other types of enterocolitis, such as bacterial enterocolitis, with high specificity. The undiagnosed patients who were prescribed antibiotics before IBD diagnosis were not still definitely excluded in the current retrospective study design, especially because CD patients often suffer from perianal fistula before diagnosis. A detailed prospective study will likely be required to elucidate the short-time impact of antibiotic use and to conclude the risk of antibiotic use in CD development without reverse causality. Another limitation of the work is that information regarding the prescription of drugs was provided, but it was impossible to confirm whether they were actually administered. Most patients take prescribed drugs; however, some who are worried about side effects or whose symptoms have improved may avoid taking new drugs. This study did not have information on these aspects. The current study also lacked information regarding the risks of developing IBD, such as smoking, appendectomy, or familial history of IBD.

It has been clinically shown that some antibiotics are useful in treating active CD^28–30^, and reducing the risk of a flare-up^31^. On the other hand, antibiotics have little effect on UC^32,33^. One potential mechanism that may explain the effectiveness of antibiotics in the treatment of CD inflammation may be the prevention of pathogenic bacterial growth. However, “normal luminal microorganisms” may be disturbed by antibiotic use prior to IBD diagnosis. Thus, pathogenic bacteria may have the opportunity to grow and adhere to the intestines of genetically susceptible individuals. The effect of antibiotics in the intestinal immune system influenced by luminal microbiota at pre- and post-onset of IBD remains unclear and should be further studied.

## Conclusion

Our study determined the risk of antibiotic use and enterocolitis independently in the development of CD and UC. As observed in populations of western countries, dysbiosis produced by these environmental factors influences the onset of UC and presumably CD in Asian populations. Further studies should assess the role of “normal luminal microorganisms” in IBD development and determine unique characteristics of individuals susceptible to IBD for whom antibiotic use and enterocolitis promote chronic intestinal inflammation.

## Methods

### Objectives and evaluation

The primary objective of this study was to evaluate the association between prescription of antibiotics or incidence of enterocolitis with subsequent development of IBD. The secondary objective was to evaluate the risk of the number and the type of antibiotics, the age, and the identification of specific bacterial pathogens for enterocolitis.

### Data sources

Population-based administrative data used in the study were collected from the Japan Medical Data Center (JMDC) health insurance claims database. Over 7 million people who have belonged to the Japan Health Insurance Association from 2005 have been included in this database. The JMDC database contains medical and prescription claims with diagnoses coded using the tenth revision of the international statistical classification of diseases and related health problems (ICD-10) classification and the Japanese standard disease code. Drug prescription information was coded using the Anatomical Therapeutic Chemical (ATC) classification system, which included generic drug information and a description of clinical procedures defined with the Japanese standardized procedure code. Hospitalization, outpatient, and prescription data from the Japan Health Insurance Association was provided. Demographic information, including sex and age was also obtained from the database. The database assigned each insured individual a unique identification number, and information regarding diagnoses and prescriptions provided in any hospitals or clinics are available. Personally identifiable information, however, is not available in the database.

### Study populations and methodology

Patients with a new diagnosis of IBD between January 2015 and December 2018 and identified using an ICD-10 classification; CD (K-50); and UC (K-51) were enrolled. Eligibility of each IBD diagnosis was ascertained by following up the diagnosis for one year without change of diagnosis code and no IBD diagnosis code for 24 months prior to the first diagnosis code. Patients who had previously been diagnosed with IBD were excluded.

For the control, for each CD and UC patient, five temporal- (during the same calendar year), age- and sex-matched non-IBD subjects with an unrelated diagnosis to IBD were randomly sampled. The date of IBD onset was defined as the first documentation of an ICD-10 diagnostic code. Enterocolitis which included just colitis such as infectious colitis with *Escherichia coli*, *Campylobacter*, or *Clostridioides difficile*, also included bacterial nonspecific gastroenteritis, was designated with ICD-10 codes A02, A04-A09, and A56. ICD-10 codes and specific diagnoses are shown in Supplementary Table 1. Drugs prescribed were identified using the following ATC codes: J01-04 and A07A for antibiotics and antibiotic type were identified as tetracyclines, penicillins, cephalosporins, carbapenems, TMP/SMXs, macrolides, aminoglycosides, quinolones, metronidazoles, anti-fungal agents, or “others” (Supplementary Table 2). These “others” included glycopeptides, polymixins, fosfomycins, linezolids, fidaxomicins, rifaximins, and anti-mycobacterials.

Prescription of antibiotics or incidence of enterocolitis for 1 and 2 years before the diagnosis of IBD was assessed for evaluating the risk of subsequent development of IBD.

### Latent period

The period in which patients were prescribed with antibiotics or diagnosed with nonspecific enterocolitis within a 3-month time frame before their diagnosis of IBD were excluded from this study for analyzation to minimize the potential for reverse causality.

### Ethical considerations

As the data provided from the JMDC database were anonymous, informed consent was not required based on ethical guidelines for epidemiological research issued by the Japanese Ministry of Health, Labour, and Welfare. The protocol for this study was examined and approved by the Ethics Committee of Akita University School of Medicine (approval number: 2819). All the methods were performed in accordance with relevant guidelines and regulations.

### Statistical analysis

Conditional logistic regression models were used for this case–control study^34^. AORs and 95% CIs between cases and their age- and sex-matched controls were reported after adjusting for previous antibiotic use and the presence of enterocolitis. The diseases unrelated non-IBD populations were fitted examining CD and UC separately. Control participants for CD and UC were combined when determining the odds ratio of all IBD patients. According to the number of antibiotics prescribed, antibiotic users were divided into three groups as follows: 1, 2, or 3 and more than 3 antibiotics. AORs and 95% CIs adjusted for age, sex, and either the number of prescribed antibiotics or antibiotic type were determined using logistic regression models. Enterocolitis was divided into bacterial pathogen-specific and nonspecific groups. Thereafter, logistic regression analyses were performed individually. All analyses were performed using EZR software (Saitama Medical Center, Jichi Medical University, Saitama, Japan)^35^.

## Supplementary Information

Supplementary Information.

## Abbreviations

IBD: Inflammatory bowel disease
CD: Crohn’s disease
UC: Ulcerative colitis
CI: Confidence intervals
JMDC: Japan medical data center
ICD: International statistical classification of diseases
ATC: Anatomical therapeutic chemical
TMP/SMXs: Trimethoprim/sulfamethoxazoles
AORs: Adjusted odds ratios
SD: Standard deviation
